# The nutritional feed gap: Seasonal variations in ruminant nutrition and knowledge gaps in relation to food security in Southern Africa

**DOI:** 10.1007/s12571-024-01509-1

**Published:** 2024-12-23

**Authors:** Andrew S. Cooke, Honest Machekano, Lovemore C. Gwiriri, Jonathan H. I. Tinsley, Gleise M. Silva, Casper Nyamukondiwa, Andrew Safalaoh, Eric R. Morgan, Michael R. F. Lee

**Affiliations:** 1 Department of Life Sciences, School of Natural Sciences, College of Health and Science, University of Lincoln, Lincoln, UK; 2 Department of Biological Sciences and Biotechnology, Botswana International University of Science and Technology, Palapye, Botswana; 3 Department of Zoology and Entomology, University of Pretoria, Pretoria, South Africa; 4 Centre for Agroecology, Water and Resilience, Coventry University, Coventry, UK; 5 Department of Zoology and Entomology, Rhodes University, Makhanda, South Africa; 6 Department of Animal Science, Lilongwe University of Agriculture and Natural Resources, Lilongwe, Malawi; 7 Institute for Global Food Security, Queen’s University Belfast, Belfast, UK; 8 Department of Agricultural, Food and Nutritional Science, University of Alberta, Edmonton, Canada; 9 School of Sustainable Food and Farming, Harper Adams University, Edgmond, UK

**Keywords:** Livestock, Sustainability, Food security, Animal nutrition, Animal health, Goat, Smallholder

## Abstract

Livestock production is critical to food security and rural livelihoods across Southern Africa. Despite progress in livestock science research in recent years, the seasonal availability and quality of feed remains one of the key challenges to livestock productivity in Southern Africa. In particular, dry weather conditions, the lack of rain and lower temperatures in the dry season cause herbaceous plants to die back and browse species to defoliate, limiting the abundance, quality, and variety of feed available. This creates a ‘Nutritional Feed Gap’, defined here as the combined effect of the sharp reduction in both forage quantity and quality from the wet to the dry season and the risk that it poses to ruminant production systems and the food security of the people and communities reliant on them. Understanding the nature and extent of how seasonality impacts ruminant production potential can thus contribute towards mitigating negative impacts of extreme weather and climate change on food systems. In this review, we characterise this nutritional feed gap in terms of forage abundance and nutrition as well as discussing how climate change may shape the future nutritional landscape. Whilst some forage nutrient concentrations varied little by season, crude protein and phosphorus were consistently found to decrease from the wet season to the dry season. We also identify a shortfall in primary research that assess both forage quality and quantity simultaneously, which forms part of a broader knowledge gap of our limited understanding of the impact of limiting factors to ruminant production on short and long-term food security across Southern Africa.

## Introduction

**Fig. 1:**
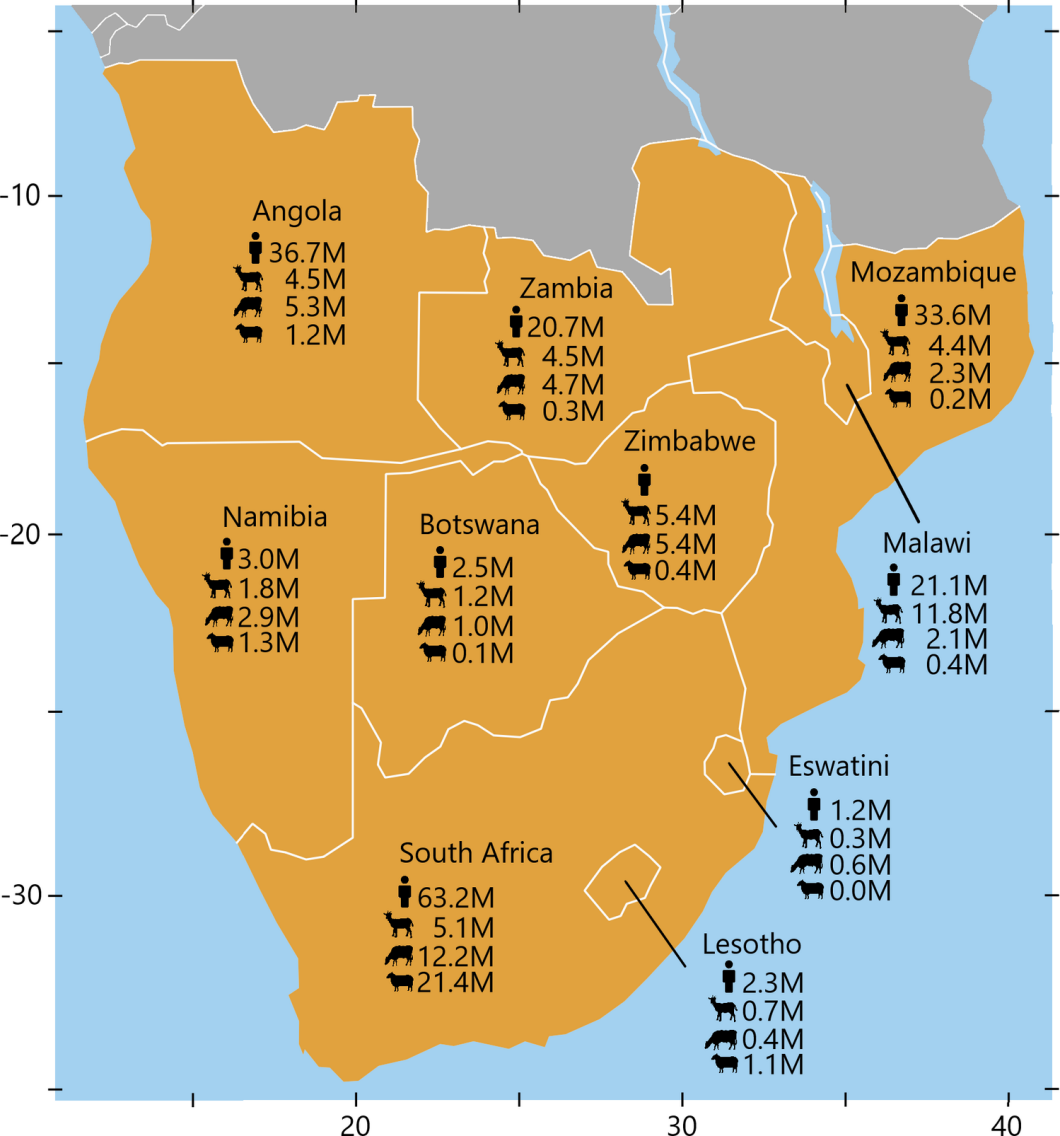
Map of countries in Southern Africa (SoAf) as defined for this study. Axes are latitude and longitude. Human (top), goat (top-mid), cattle (bottom-mid), and sheep (bottom) populations are included (FAO, 2022b; The World Bank 2022a). Greyed-out countries are not considered part of SoAf for the purpose of this review. Map created using QGIS (2022)

Southern Africa (SoAf) (Fig. 1) is home to approximately 190 million people, half of whom live in rural communities (FAO, 2022a). Across the region, many rural households own and rear livestock, particularly ruminants, to support their food and financial security. Regionally, there are approximately 40 million goats, 37 million cattle, and 26 million sheep (FAO, 2022b). However, due to the socio-economic value assigned to ruminant livestock, social stratification in livestock ownership is prevalent. Poorer and food insecure households are more likely to own small herds of small ruminants (goats or sheep), than larger ruminants (cattle) (Airs et al., 2023; Gwiriri et al., 2023; Taruvinga et al., 2022). Ruminant populations are not distributed uniformly but are instead driven by socio-economic and geographical factors. For example, Malawi has a particularly high density of ruminants, reflecting its high human population density (FAO, 2022b). Comparatively, Botswana has the lowest ruminant density and second lowest human population density, in part due to its expansive national parks and the Kalahari Desert (FAO, 2022a). In South Africa, large herd sizes of cattle and sheep are owned by relatively resource-endowed households who engage in commercial livestock marketing. Taruvinga et al. (2022) reported that in South Africa, household income, age, gender, and employment status were important predictors of livestock species ownership at the household level.

### Regional seasonality

**Fig. 2:**
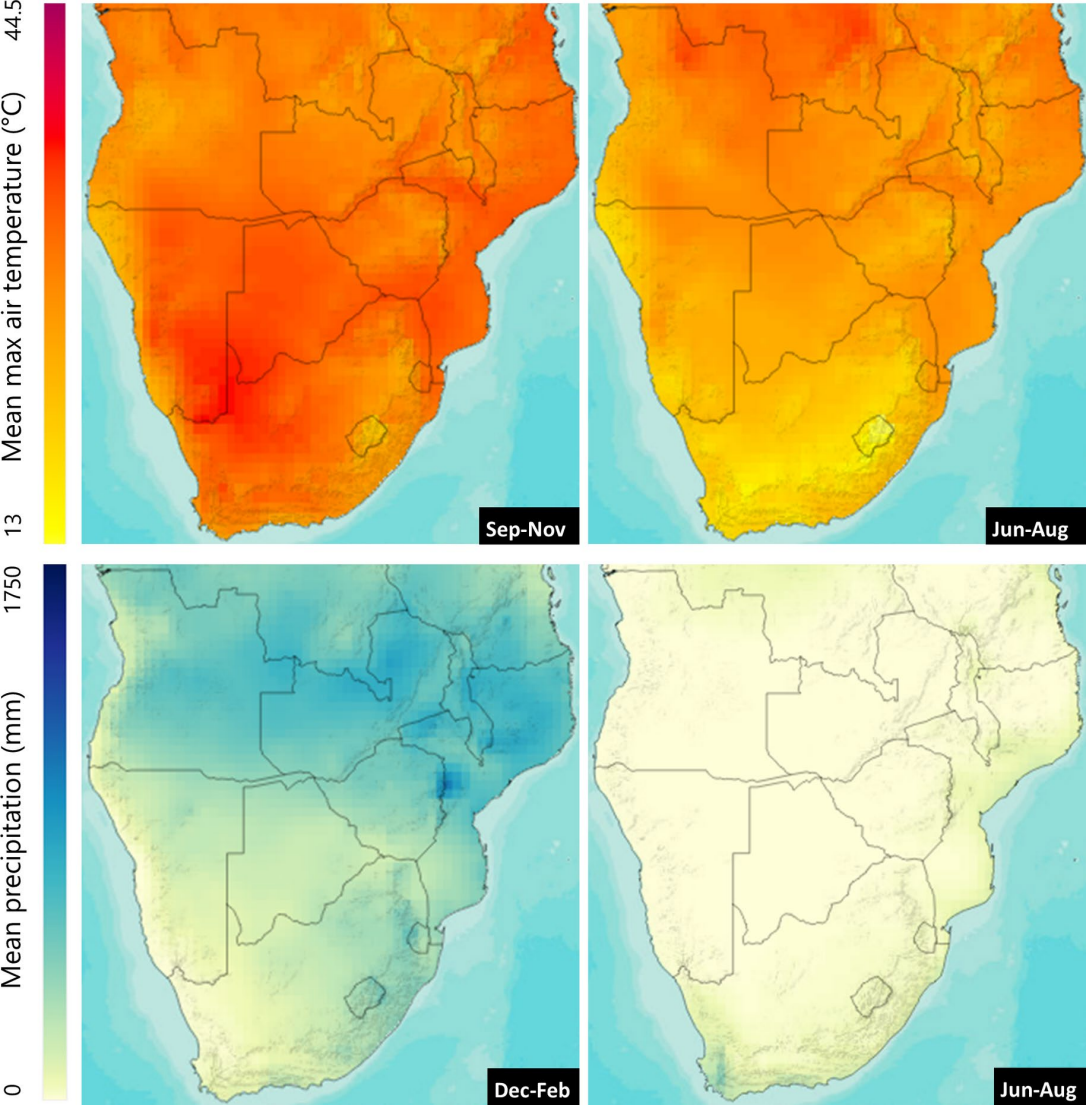
Mean maximum air temperature (1991–2020) for the hottest quarter of the year (Sep-Nov) and coolest quarter of the year (Jun-Aug). Mean precipitation (1991–2020) for the wettest quarter of the year (Dec-Feb) and the driest quarter of the year (Jun-Aug)

There are two distinct seasons in SoAf, the hot and wet (‘wet season’) and the cold and dry (‘dry season’). The seasonal calendar varies somewhat based on exact geographical location, topography, and microclimates (Fig. 2). Broadly, the wet season runs from October/November to March/April, during which more than 95% of annual rainfall may occur, typically in short but frequent events. Rainfall is spatially variable, for example, the south-west of the Namibian desert may receive < 50 mm per annum, compared to 650 mm in the north-east and greater rainfall elsewhere in SoAf such as Zomba, in Malawi, which may receive 1500 mm. The warmth and moisture of the wet season allows for fresh vegetative growth. Grasses, forbs, and other herbaceous species can flourish, and hardier browse species (trees and shrubs) foliate. The dry season, from mid-April to September/October, is a period of drought with little to no rainfall for long periods. Notably, water scarcity is a significant limiting factor to grassland productivity. Soft-stemmed herbaceous species die back, leaving barren ground. Browse species stop producing new green growth and often defoliate. This seasonality has an impact on the native flora and the livestock which depend on them for nutrition. The potential of any feed to support animal production depends on the quantity consumed and the extent to which the feed consumed supplies nutrients to meet the requirements (maintenance + performance) of the animal. Schneiderat et al. (2005) reported that the impact of droughts in 2003 in central Namibia led to significant shortfalls in forage biomass, metabolisable energy (ME) and crude protein (CP) relative to the requirement for livestock. It has been observed that feed intake rates fall significantly from the wet season to the dry season (Dziba et al., 2003). This is a constraining factor in livestock production and something farmers are aware of (Lamega et al., 2021). The extreme seasonality, exacerbated by the impact of climate change, in SoAf, presents a significant challenge to animal nutrition on two main fronts. Firstly, a reduction in forage availability may limit dry matter intake (DMI) and consequently the quantity of nutrients available to animals for health and performance. Secondly, the quality of nutrition may decrease as plants die back or defoliate, meaning that diet compositions are sub-optimal or potentially deficient in the nutrients required for ruminant health and performance. There is limited research linking those two aspects in SoAf explicitly, with research typically focussing on either forage quantity or quality, but seldom both.

### Food security and nutrition

**Fig. 3:**
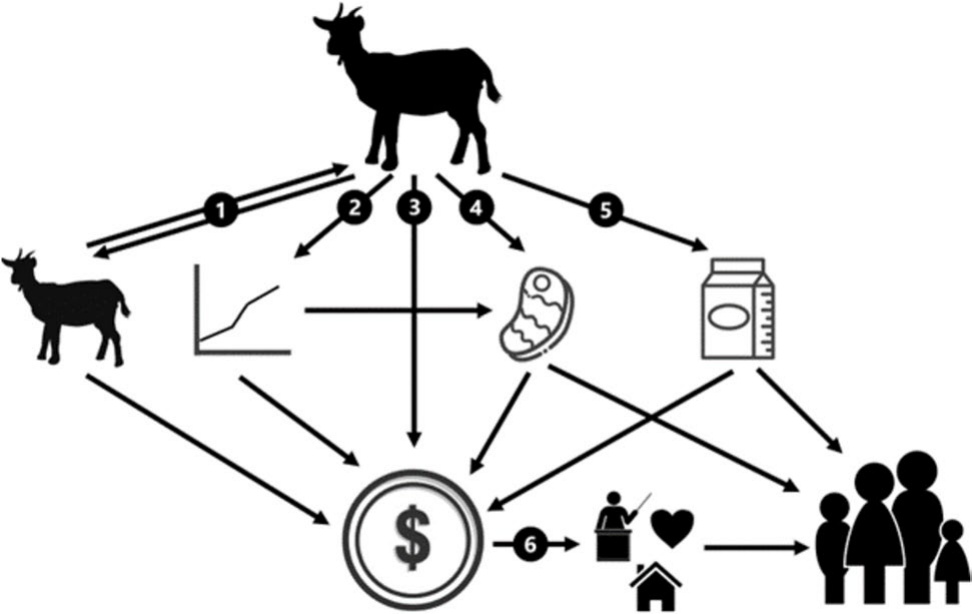
Summary of the various roles that goats play in households and communities. (1) Goats produce offspring which can either be sold or kept. (2) Growing goats may increase in financial value, and nutritional value as they gain more liveweight. (3) Goats can be sold at any time to meet demands for cash. (4) Goat meat can be sold or consumed. (5) Goat milk can be sold or consumed. (6) Financial revenue from goat enterprise can be used to fund activities such as education, and healthcare, and contribute to general household finance

Ruminants play a significant role in the livelihoods of individuals, households, and communities, providing nutritional, economic, and socio-cultural benefits (Fig. 3). Across the region, production of and access to animal products is widely considered to be a key component of food security (Danso-Abbean et al. 2024; Herrero et al., 2014; Smith et al., 2013). Livestock systems underpin food security directly, in terms of meat and milk production, and indirectly through income generation (Eik et al., 2008). In rural areas, where food insecurity is most prevalent, goats are particularly important. Kaumbata et al. (2020) reported that, in Malawi, goats accounted for more than 60% of total livestock household-based income compared to 17.6%, 15.5% and 4.1% for cattle, pigs and chickens, respectively. The utilisation of goats varies by farmer and immediate circumstance. Khowa et al. (2023) reported, from South Africa, that 66% of rural farmers slaughtered goats for meat, whilst this figure was 48% for peri-urban farmers. The use of goats for ‘urgent sale’ was high across both groups (82% rural, 73% peri-urban. Ultimately, the returns on ruminant systems are variable and with that comes the flexibility for owners to realise different benefits based on their needs. For example, when times are plentiful and food security is high, ruminants may be used for cash to support education. But in a time of famine and low food security, the ruminants may be used for their meat.

The ability of ruminants to consume (often freely available) forages unsuitable for human consumption and convert them into milk, meat and cash provides these benefits. Ruminants are typically reared in mixed cropping systems, where they can consume crop residues and return manure to fertilise the soils (Mataveia et al., 2021). The diversification of crop and livestock systems also provides added resilience against unexpected events such as droughts, pests, and disease, which may impact one aspect of a system more than another. The ability of ruminants to convert natural capital to nutritional and financial value is especially important in SoAf, which suffers from high rates of malnutrition, food insecurity, and poverty. An estimated 104 million people live in a state of food insecurity, of which 50 million are under ‘severe food insecurity’ and 22 million are undernourished (FAO, 2022c). This includes high rates of micronutrient deficiencies (hidden hunger) (Gebremedhin, 2021; White et al., 2021), many of which can be obtained from animal products (Adesogan et al., 2020). Vitamin B12 which is predominantly acquired through the consumption of animal products, is recorded as having extremely low intake rates in the region (Table 1). A similar trend was observed for zinc, with recorded deficiency rates ranging from 18% in Eswatini to 45% in Zimbabwe (Ritchie & Roser, 2017).

**Table 1:**
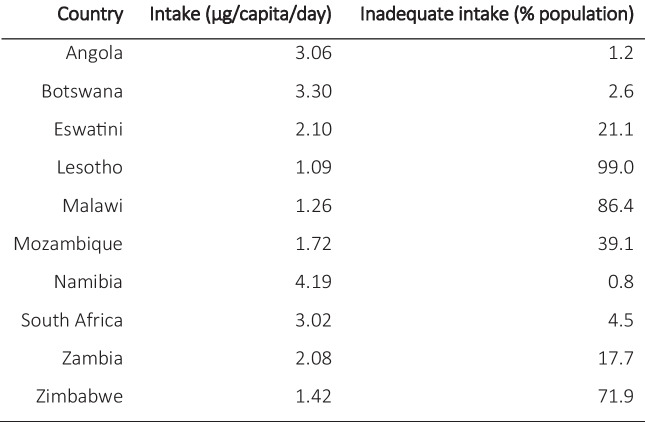
Intake rates of vitamin B12 (ug/capita/day) and rate of inadequate intake (%) across SoAf countries in 2017. Data taken from Gebremedhin (2021). µg = micrograms

Malnutrition is not only a direct risk to individual health, but has secondary impacts such as increased susceptibility to disease, growth stunting, and reduced educational performance (Adebisi et al., 2019; Adesogan et al., 2020; Fan et al., 2022), which themselves may impact the long-term food security of individuals (De Cock et al., 2013). These factors are not only consequential to the individual but have broad socio-economic impacts when scaled to community and regional levels. The ability of healthy and productive animals to mitigate these nutritional shortfalls is therefore beneficial to both individuals and wider society. The mean poverty rate across SoAf is 42% (The World Bank 2022b) and is especially high in rural areas where livestock ownership is most common. Live animals, meat, and milk are saleable commodities which can generate an income stream for households. Income can then be used to support crucial activities such as education, healthcare, and investment. Surveying farmers in South Africa, Kunene & Fossey (2006) reported that 79% of goat sales were to meet an immediate need for cash, such as school fees, comparable to results by Enkono et al. (2013) who reported, from Namibia, that 86% of cattle sales were to pay school fees (42%) or provide cash for household activities (44%). This is also reflected in research by both Bolowe et al. (2022) and Monau et al. (2017) who reported that needs-based cash value was the primary reason for goat ownership in Botswana. Similarly, Khowa et al. (2023) reported that 60% of farmers in KwaZulu-Natal (South Africa) kept goats for a combination of subsistence use and sales. Live animals also function as tangible assets, as a store of both financial and nutritional value (Kaumbata et al., 2020). This can be seen as a form of insurance and banking. In times of financial need, individuals can sell animals for cash, which may be used to cover costs such as medical expenses or school fees. In times of exceptional food insecurity animals can either be sold or exchanged for staple grains or be slaughtered to provide high-quality nutrients to help alleviate the issue.

Where ruminants do not directly contribute to household food security (e.g. when sold for cash for non-food purposes), they still contribute to community and regional food security by supplying the market place. Indeed, the meat markets in the region, especially in poorer and rural communities, are predominantly supplied by smallholders. Bai et al. (2020) reported that, in Malawi, seasonal fluctuations in the cost of animal products were not as large or significant as those for other foods, though the reasons behind this and whether it is widely common, is unclear. Zant (2023) studied long-term livestock and meat prices in Malawi in response to drought, reporting that livestock sales increased during droughts, to generate cash for purchasing other food. Interestingly, this reduces the value of meat comparative to maize, but predominantly due to an increase in maize price. This highlights both the comparative stability of meat prices and the role it can play in supporting the food security of households during difficult times.

### Objective

The primary objective of this review was to investigate the patterns and extent of the seasonal fluctuations in forage nutrition and forage availability available for ruminant production in SoAf, resulting in the characterisation of a “Nutritional Feed Gap”, and pertinence of that within food security. This was achieved through a non-systematic, but exhaustive, literature search using Google Scholar, Web of Knowledge, and Google. The focus of the search was on finding research that reported seasonal data on forage quality and/or quantity from any country or countries within SoAf. Initial search terms generally included an object (e.g. “*forage*”*,* “*plant*”, “*[species name]*”, a metric/measure (e.g. *“quality*”, “*nutrition*”, “*availability*”), a location (e.g. *“Malawi*”, “*Southern Africa*”, “*Africa”*), both with and without a temporal component (e.g. *“seasonality*”, “*temporal*”)*,* However, search terms evolved organically based on researchers´ judgement. For the creation of tables, included papers had to present numerical data on forage quality (nutrition) and/or quantity at individual species level, for a species suitable for consumption by ruminants, for at least one time point in both the wet and dry seasons. This was non-time-bound and whilst it predominantly included primary peer-reviewed research, indexed on major repositories, it also included some ‘grey’ literature, for example, a PhD thesis. The reference lists of identified works were also examined for potential work that was not found via the primary searches. Searches were conducted in English and in Portuguese.

Climate change, intervention, and areas for future research were also discussed.

## Forage availability

**Fig. 4:**
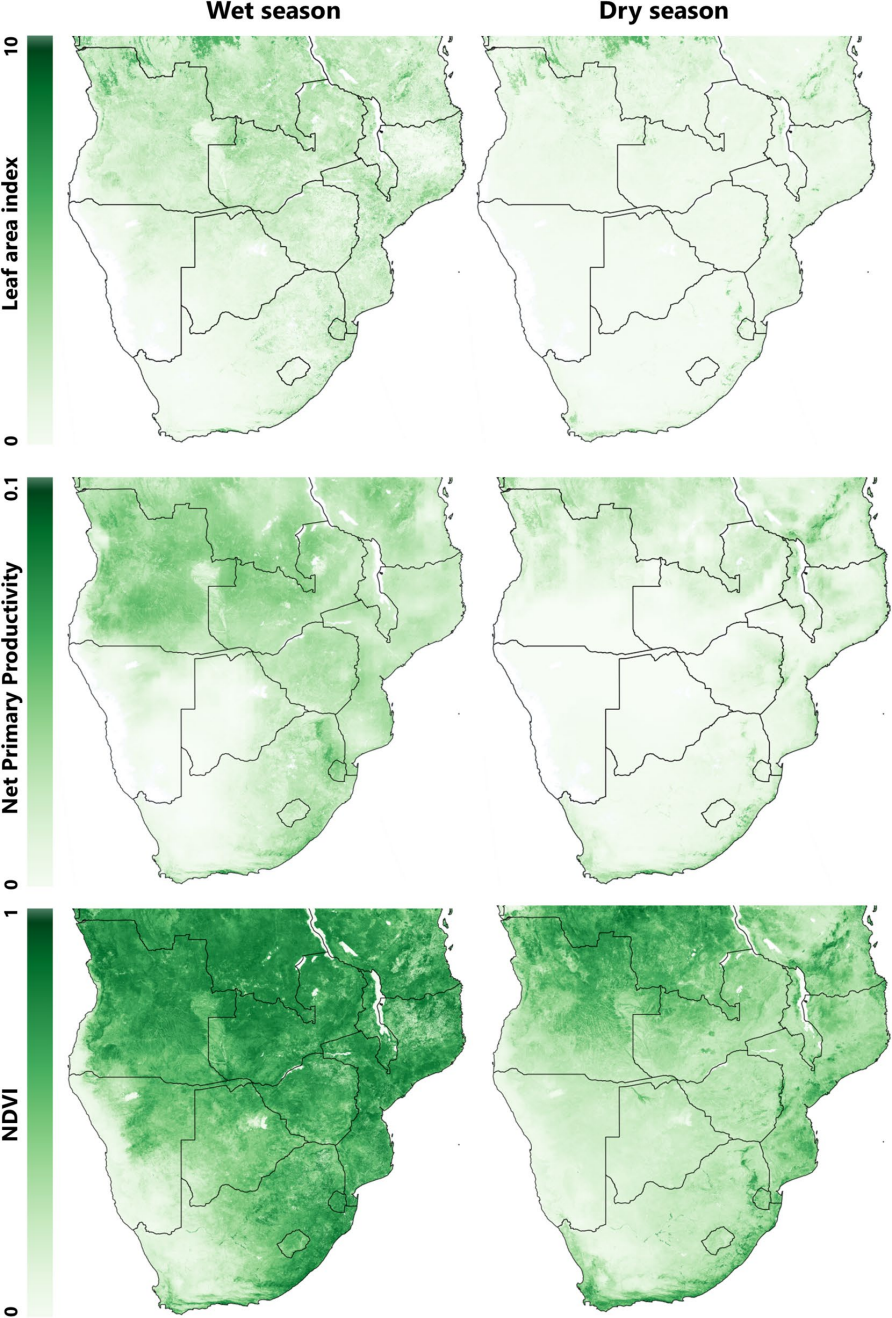
Remote sensing imagery contrasting the wet and dry seasons in SoAf. Top: Leaf area index average across 8-days in the wet season (10/02/23 to 17/02/23) and the dry season (29/08/23 to 05/09/23). Middle: Net primary productivity for 8-days in the wet season (10/02/23 to 17/02/23) and the dry season (14/09/23 to 21/09/23). Bottom: Normalised difference vegetation index (NDVI) for 16-days in the wet season (02/02/23 to 17/02/23) and the dry season (14/09/2023 to 01/10/2023). Data taken from NASA Earthdata (NASA, 2022)

Changes in forage availability can be considered both in terms of absolute quantities available and the diversity of forage species available, though it is the combination of these factors which is important for ruminant health and the system’s carrying capacity. At a regional level, seasonal vegetative biomass differences are most clearly visible by remote sensing imagery such as of leaf area, net primary productivity, and normalised difference vegetation index (NDVI) (Fig. 4), which highlights the stark seasonal differences in the abundance of vegetation across time and space. The southwest of the region, particularly southern Namibia and western South Africa has low (< 0.5) NDVI year-round and during the dry season, this reduces further and extends north and east across Botswana and into Zimbabwe and Zambia. Differentiating between browse and grazing/herbaceous plant availability is preferential. Kahiu et al. (2024) explored livestock mortality models that utilised forage indexes and found that the best fits were obtained by distinguishing browse and grazing/herbaceous plants.

Studying rangeland pastures in Mozambique, Muir & Alage (2001) reported a mean dry matter yield of 5209 kg ha^−1^ at the end of the wet season compared to 3822 g ha^−1^ at the end of the dry season. Similarly, in Botswana, Mphinyane et al. (2015) observed biomass availability to be 5130 kg ha^−1^ at the end of the wet season but just 2400 kg ha^−1^ at the end of the dry season. This reduced availability of forage exerts an effect on goat diets. In Botswana, Omphile et al. (2005) reported that the herbaceous species made up approximately 31% of goats' diet in the wet season but just 5% in the dry season. However, browse species made up 41% in the wet season, increasing to 65% in the dry season – seemingly compensating for the shortfall in herbaceous forage availability. Similarly, in Malawi, Becker & Lohrmann (1992) reported that the feeding time (% of total) of grasses and herbs reduced from 54% in the wet season to just 6% in the dry season, meanwhile browse feeding increased from 46 to 93%. This was driven by a reduction in the cover of grasses and herbs (67% to 15%) whilst the ‘fresh’ tissue of browse species dried out but remained available.

Omphile et al. (2005) reported that these effects lead to a decrease in the diversity of plants consumed by goats, characterized by increased consumption of browse to compensate for the lower abundance of grasses and forbs. This significantly increased the overlap of goat diets with those of sheep and cattle, highlighting the potential for competition in accessing resources. Whilst a seasonal shift in diet towards browse species is suitable for goats, these are less suited to cattle and sheep. Bennett et al. (2007) investigated the grazing preferences of cattle and sheep in South Africa, finding that during the dry season, they chose to forage crop residues from arable land over grassland. Although not studied directly in SoAf, evidence from Kenya suggests this could create competition for crop residues that have value as mulch (Baudron et al., 2014), especially where manure may not necessarily be returned to the soil, as it is also valued as a fuel source when dried.

## Forage nutrition

Forage quality underpins health and productivity in ruminants which, in turn, supports sustainable and productive food systems and security. Animals suffering from nutrient deficiencies may have reduced growth, lower fertility, and increased susceptibility to disease (Hidiroglou, 1979; NRC, 2000, 2006). For animals without specific deficiencies, sub-optimal feed concentrations of ME and CP will yield sub-optimal performance, which consequently may mean less meat, milk, and value for the owner (NRC, 2000, 2006). Highly fibrous forages, especially those with high lignin contents, act as ‘gut-fill’ which may make the animal feel sated, but without adding much nutritional value. In periods of scarcity, however, such feed may be crucial for ruminants to fuel rumen fermentation and meet maintenance requirements, providing a vital asset for resource-poor farmers. The nutritional components are evaluated in detail below.

### Energy

Across the literature, the reporting of forage energy content is inconsistent and can be reported as gross energy (GE), digestible energy (DE), and metabolisable energy (ME). Furthermore, these are rarely measured directly and often derived from prediction equations that can be of low accuracy and/or not necessarily derived from data of the same plant species or type.

Only five studies were found containing seasonal data for forage energy content, possibly due to the challenges of quantifying energy content. This limits the degree to which conclusions can be drawn from this, but highlights a specific knowledge gap in the field of forage nutrition in the region. Forage energy concentrations typically reduced from the wet season to the dry season (Table 2), with reductions seemingly greatest in grass species, compared to browse (Chiphwanya et al., 2017; G. A Mataveia, 2019; Müller et al., 2021; Ravhuhali et al., 2022). Chiphwanya et al. (2017) reported GE contents of grasses, during the dry season, far lower than might be expected, reporting 10.2 MJ kg^−1^ DM for *Panicum maximum*. For the same sample, an NDF content was reported at 882 g kg^−1^ DM (which appears unexpectedly high) and with carbohydrates having GE concentrations of approximately 16–17 MJ kg^−1^, this does bring into question the validity of their results.

**Table 2:**
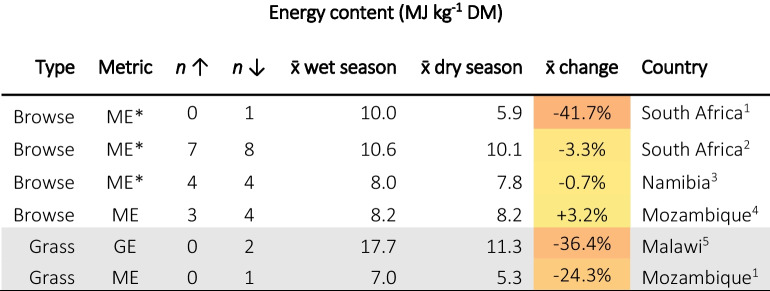
Summary of seasonal changes in energy contents of forages in SoAf. The third (n↑) and fourth (n↓) columns represent the number of species that increased or decreased in concentration. The fifth (x̄ wet season) and sixth (x̄ wet season) columns are the mean values reported for the wet season and dry season, respectively. The seventh column (x̄ change) is the average of the change for each reported species between the wet and dry seasons and is therefore not the same as the difference between the means of *all* species reported in columns five and six. The colouration of cells is proportional to the percentage change with + 100% being dark green and −100% dark red. *Represents data converted from Mcal to MJ by a factor of 4.184. Superscripts after countries are citations as follows: 1. Müller et al. (2021), 2. Ravhuhali et al. (2022), 3. Rees (1974), 4. Mataveia (2019), 5. Chiphwanya et al. (2017)

### Fibre

As the dry season progresses, foliage cover reduces and fresh growth may slow or cease. For browse, this means that woody trunks and branches remain, which are high in fibre and lignin. Grasses and herbaceous species may die back and the parts that survive are likely to be the older, thicker, straw-like stems, which will be more fibrous than lusher material would be. Indeed, the literature shows a general trend of moderate increases in neutral detergent fibre (NDF), acid detergent fibre (ADF), and acid detergent lignin (ADL) from the wet to dry season (Table 3). As previously mentioned, the high NDF content reported by (Chiphwanya et al., 2017) is to be taken cautiously. Many of the browse species found across SoAf are reportedly rich in tannins, this poses a challenge to fibre analysis as tannin-bound proteins are insoluble in acid detergent. This can lead to incorrectly high ADF results (sometimes in excess of NDF results, which would not be possible) (McArthur, 1988). Though there was no evidence of this in the papers included in this review, it is something to be wary of when interpreting the literature.

**Table 3:**
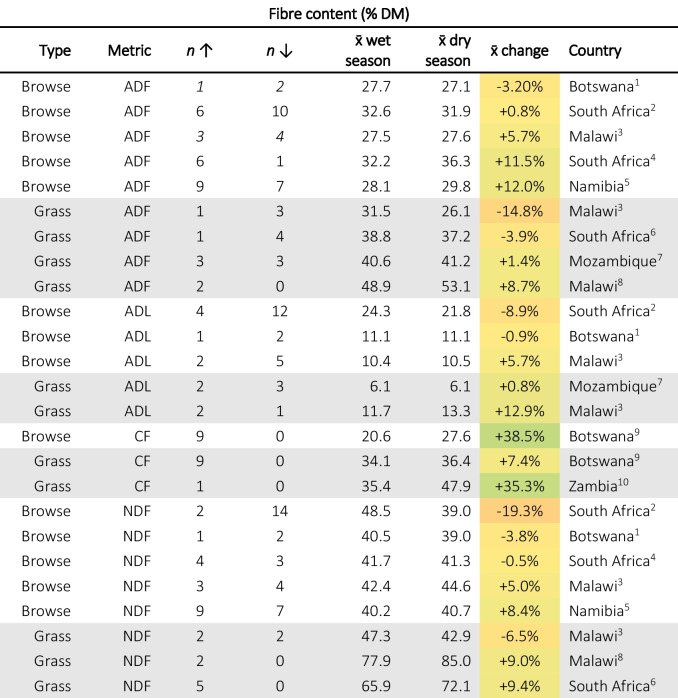
Summary of studies that investigate seasonality in forage fibre fractions. The third (n↑) and fourth (n↓) columns represent the number of species that increased or decreased in concentration. The fifth (x̄ wet season) and sixth (x̄ wet season) columns are the mean values reported for the wet season and dry season, respectively. The seventh column (x̄ change) is the average of the change for each reported species between the wet and dry seasons and is therefore not the same as the difference between the means of *all* species reported in columns five and six. The colouration of cells is proportional to the percentage change with + 100% being dark green and −100% dark red. Superscripts after country names are citations as follows: 1. Cooke et al. (2024), 2. Ravhuhali et al. (2022), 3. Cooke et al. (2023), 4. Naumann et al. (2017), 5. Marius et al. (2021), 6. Ayanda et al. (2013), 7. Muir and Alage (2001), 8. Chiphwanya et al. (2017), 9. Mphinyane et al. (2015), 10. Siulapwa et al. (2016)

### Protein

Dietary protein concentrations below 6–8% can depress ruminant voluntary feed intake and digestibility due to the reduced function of fibrolytic bacteria (Lazzarini et al., 2009; Pugh, 2014). Higher protein concentrations are generally beneficial to growth and performance, though this peaks at 15–18% DMI, above which additional benefits are minimised or lost reducing nitrogen use efficiency through a higher rate of nitrogen excretion (NRC, 2000, 2006; Salah, 2015).

Protein contents in herbaceous species are typically lower than those of browse species, though both show a trend of declining protein concentrations from the wet season to the dry season (Table 4). Seasonal CP reductions were especially prominent in grasses, with reductions often in excess of 30%, meanwhile, reductions in browse species were still prevalent, though less extreme. There were, however, notable exceptions. Studying the seasonal concentrations of cultivated grasses under fertiliser applications, Muir and Abrao (1999) reported increases in CP for seven of nine grasses. With regards to browse species, Marius et al. (2021) reported large CP increases from the wet season to the late dry season for three species in particular: *Colophospermum mopane* (2.6% to 8.1%), C*ombretum collinum* (3.1% to 7.9%), and *Grewia bicolor* (2.4% to 11.9%), characterised by especially low concentrations in the wet season. The reason for this is not entirely clear, though it may be due to sampling methods as Marius et al. (2021) exclusively collected leaf samples whilst others typically included stem. Certainly, elsewhere, wet season CP concentrations of *Grewia bicolor* have been reported as far higher, often around or above 15% (Aganga et al., 2000; Woldemariam et al., 2012).

**Table 4:**
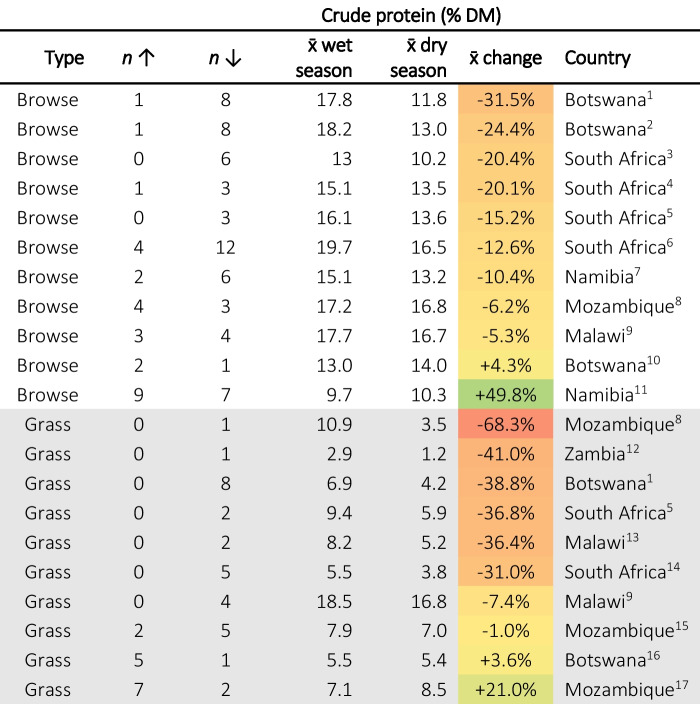
Summary of studies reporting changes in protein concentrations from the wet to the dry season. Second (n↑) and third (n↓) columns represent the number of species that increased or decreased in concentration. The fourth (x̄ wet season) and fifth (x̄ wet season) columns are the mean values reported for the wet season and dry season, respectively. The sixth column (x̄ change) is the average of the change for each reported for *each* species between the wet and dry season and is therefore not the same as the difference between the means of *all* species reported in columns four and five. The coloration of cells is proportional to the percentage change with + 100% being dark green and −100% dark red. Superscripts after countries are citations as follows: 1. Mphinyane et al. (2015), 2. Aganga et al. (2000), 3. Naumann et al. (2017), 4. Mthi et al. (2021), 5. Stapelberg et al. (2008), 6. Ravhuhali et al. (2022), 7. Rees (1974), 8. Mataveia (2019), 9. Cooke et al., (2023), 10. Cooke et al. (2024), 11. Marius et al. (2021), 12. Siulapwa et al. (2016), 13. Chiphwanya et al. (2017), 14. Ayanda et al. (2013), 15. Faftine et al. (2021), 16. Moleele (1998), 17. Muir and Abrao (1999)

It is also important to note potential differences in the protein types between browse and herbaceous species. Furthermore, protein in browse species may be less available to the rumen due to the inhibitory effects of lignin and tannins on protein bioavailability and forage digestibility, both of which are more abundant in browse species. However, in the context of ruminant nutrition in SoAf, these topics have received little attention.

### Minerals

Seasonal variations in mineral concentrations differed from mineral to mineral, some typically decrease from the wet to dry season, others increase, and others show no clear trend (Table 5). Calcium (Ca) and Phosphorus (P) showed the clearest trends. It is widely accepted that plant Ca concentrations increase during drought stress (Aliniaeifard et al., 2020) which appears to be the observed trend in SoAf, with Ca concentrations typically increasing from the wet to the dry season. However, the reported Ca concentrations across both seasons appeared to be suitable based on guidelines requirements (see: (NRC, 2006; van den Top, 2005). Conversely, P concentrations were reduced in the dry season, which has been observed worldwide as a consequence of drought stress (He & Dijkstra, 2014). Mtimuni et al. (1983), in Malawi, observed a decline in serum P concentrations of cattle from 6.4 to 4.8 mg per 100 ml with 12.7% of cattle being P deficient in the wet season increasing to 38.5% in the dry season, which aligns with changes in plant P concentrations. Studies from both Botswana and South Africa (Lukhele & Ryssen, 2003; Moleele, 1998; Mthi et al., 2021) report P concentrations below the 0.15% DM concentrations recommended by NRC (2006). Notably, Botswana and northern areas of South Africa have low soil P concentrations. This problem appears exacerbated in the dry season, likely due to a lack of water limiting the mobility of P, and potentially P uptake by plants.

**Table 5:**
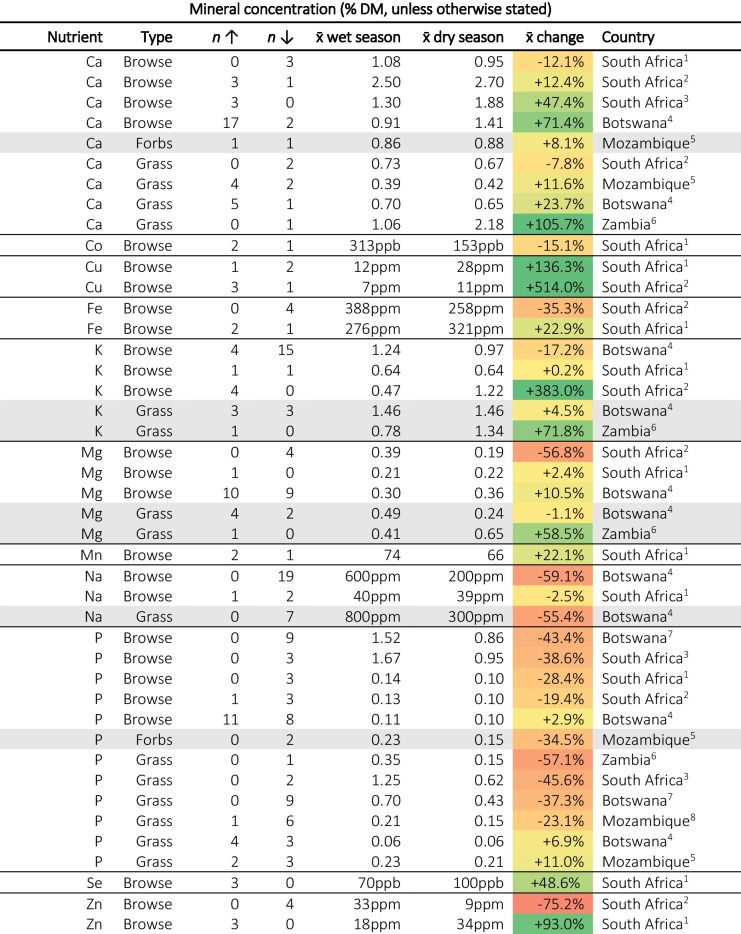
Summary of seasonal changes in plant nutrient mineral concentrations. The third (n↑) and fourth (n↓) columns represent the number of species that increased or decreased in concentration, “- “ signifies that only summary data was available. The fifth (x̄ wet season) and sixth (x̄ wet season) columns are the mean values reported for the wet season and dry season. The seventh column (x̄ change is the average of the change for each reported species between the wet and dry seasons and is therefore not the same as the difference between the means of *all* species reported in columns five and six. The colouration of cells is proportional to the percentage change with + 100% being dark green and −100% dark red. Superscripts after countries are citations as follows: 1. Lukhele & Ryssen (2003), 2. Müller et al. (2019), 3. Mthi et al. (2021), 4. Stapelberg et al. (2008), 5. Moleele (1998), 6. Faftine et al. (2021), 7. Siulapwa et al. (2016), 8. Mphinyane et al. (2015), 9. Muir & Alage (2001)

### Spatial variation

Whilst this review focuses on seasonal forage nutritional variation, it is important to be mindful of the spatial variability of factors such as climate and soil type. The region is geographically heterogeneous with large variations in elevation, climate, soil type, and coastal proximity. Such heterogeneity has implications on forage nutrition and availability. The NDVI map of the region shows lower NDVI scores in the southwest, transitioning to higher scores in the northeast, but with the southeast coastal areas of South Africa being more stable. Local and within-country variation can also be significant (Fig. 4). Beyene & Mlambo (2012) found significant differences in grass nutritional value between lower middleveld and lowveld areas of Eswatini. Similarly, Ayanda et al. (2013) found differences in yield and nutrients of grasses between highveld and lowveld grazing areas in the Eastern Cape of South Africa, though this was dependent on species and season.

**Fig. 5:**
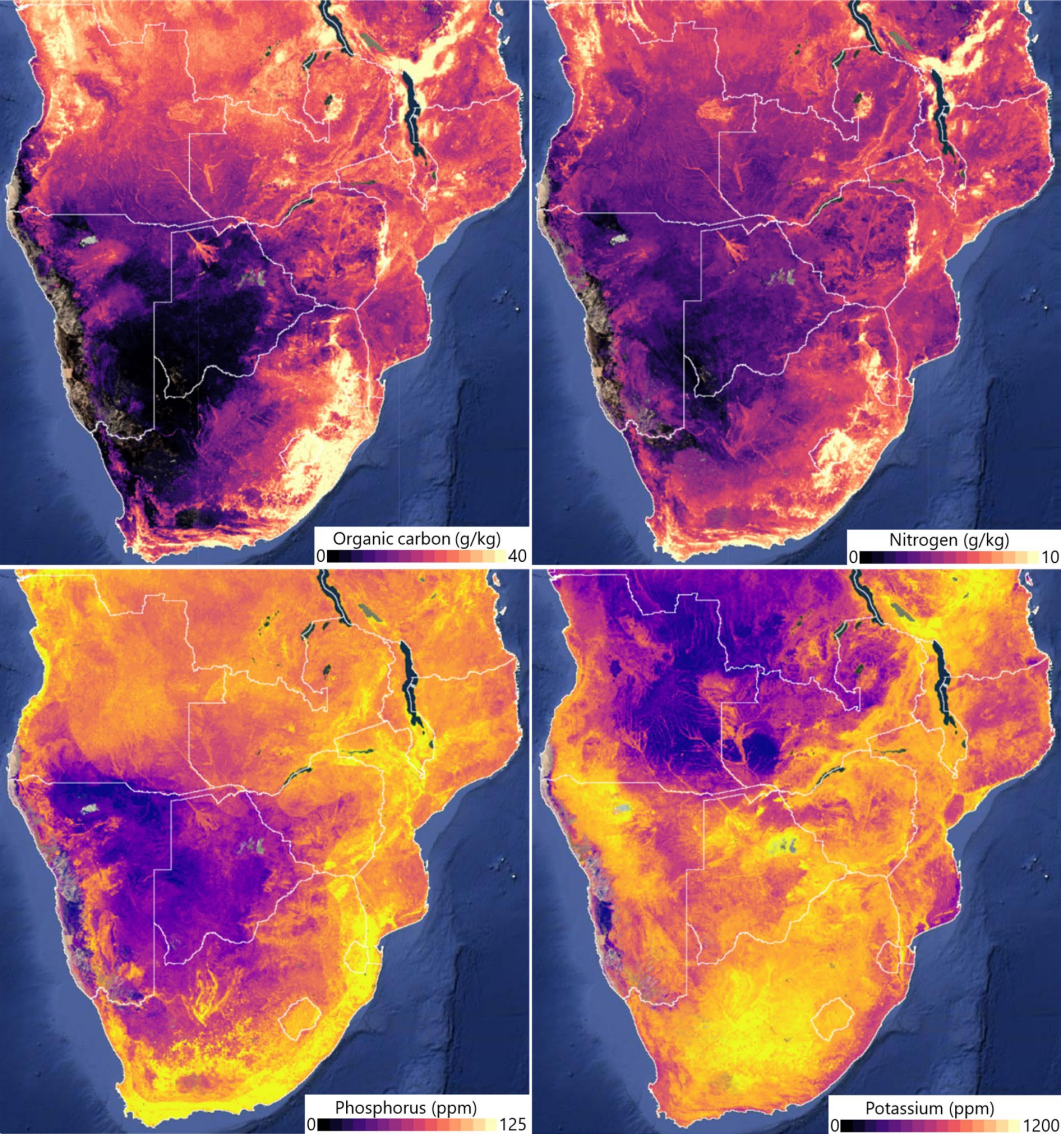
Concentrations of four key soil nutrients at depths of 0–20 cm across SoAf. Top left: organic carbon (g/kg). Top right: Nitrogen (g/kg). Bottom left: Phosphorus (ppm). Bottom right: Potassium (ppm). Data taken from the Innovative Solutions for Decision Agriculture (iSDAsoil) (Miller et al., 2021)

Soil type varies significantly across the region (Fig. 5). Soil P concentrations are acutely low across Botswana, Namibia, and parts of South Africa. Combined with the drop-off in forage P concentrations described earlier (Table 5), this is a significant concern that already low levels of P are declining further into the dry season, potentially impacting plant and animal health. Organic carbon (OC) stocks are low across the region, but especially so in the central and centre-west parts of SoAf, with low levels across large areas of Botswana, Namibia, and South Africa, notable in and around the Kalahari. Nitrogen (N) levels are also low across the region. Conversely, K levels are relatively higher across much of SoAf, though Angola and Zambia do suffer from low levels. The impact of soil type on forage nutrition is relatively well understood, with factors such as pH, organic matter, and microbial activity all influencing soil function and the supply of nutrients from soils to plants (Kao et al., 2020). However, there has been limited study of this within SoAf. Nsinamwa et al. (2005) (Botswana) found significant differences between two soil types (Hardveld [ferralsol] and Sandveld [arenosol]). Hardveld soil had significantly greater concentrations of macroelements (Ca, K, Mg, N, P) and, consequently, grasses growing on Hardveld soils had significantly higher macroelement concentrations than those growing on the Sandveld soils. Cooke et al. (2024), studying the same soil types in Botswana, found a significant difference in the ME of forages grown on the two soils, with Hardveld soils yielding plants with low ME concentrations. Differences have also been observed in South Africa. Mudau et al. (2021) conducted a detailed study of 52 browse species across two soil types, finding significant differences in concentrations of a wide array of macronutrients, amino acids, metabolites, and energy measures. Also, Havenga et al. (2004) reported on the nutritional composition of *Boscia foetida* across three locations in South Africa, finding significant differences for CP, NDF, Ca, Mg, Cu, Zn, and Mn – though soil type was not studied as a causal factor in this study. Overall, it can also be argued that in the absence of intrinsic soil type variations, other factors such as species composition and herbivore use intensity also affect forage nutrition.

### Nutraceuticals

Plant secondary metabolites, such as condensed tannins (CT), can have nutraceutical properties that may confer a degree of protection against pathogens, particularly gastrointestinal nematodes (GINs) (Machekano et al., 2023). CT extracts from browse species found across SoAf have been found to have anthelmintic properties both in vitro (Sutherland & Leathwick, 2011) and in vivo (Max et al., 2007). The anthelmintic effects of CTs are varied and not entirely understood, with indirect effects through animal resilience potentially important. Temporary binding of protein by CT, for example, can increase its bioavailability and compensate for protein losses from parasite infection (Hoste et al., 2006). Reductions in egg excretion in ruminants fed CT-enriched diets have been widely reported (Hoste et al., 2006; Max et al., 2005; Paolini et al., 2003). Effects such as reduced host mortality and liveweight have also been reported though such findings are not consistent across studies, with many finding no difference (Hoste et al., 2006).

The effects of nutraceutical availability and consumption are likely to vary seasonally with parasite challenge. Pandey et al. (1994) (for Zimbabwe) found that the prevalence of GINs in goats was greatest at the end of the wet season – with rainfall being a key driver of GIN development and transmission. The more hostile conditions in the dry season reduce the exposure of ruminants to infective GIN larvae, however, adult GINs originating from infection in the wet season can continue to survive within their host.

There are potential negative aspects to high CTs in forage: plants with high concentrations (⪆5%) can be unpalatable and can be avoided. Protein digestion may also be inhibited as CTs can bind excessively to proteins, reducing their bioavailability to rumen microbes (Koenig & Beauchemin, 2018), whilst the benefits of bypass protein might not be attained. Given the low protein concentrations of grasses in SoAf, high dietary levels of CTs (primarily from browse) may exacerbate this issue (Waghorn, 2008). Therefore, the beneficial anthelmintic properties of CTs need to be balanced against this negative – a balance which may vary based on the epidemiological and nutritional landscapes at any given time. Ruminants have been shown to actively regulate their CT intake by altering their foraging behaviour (Mkhize et al., 2018) and this may provide some in-built optimisation of CT intake.

As with other nutrients, CT concentrations vary seasonally. Naumann et al. (2017) (South Africa) reported a seasonal reduction in CT content from the wet to the dry season in 6 of 7 studies of browse species in SoAf. The mean change from wet to dry season was −16%, with extremes of −62% for *Sennegalia caffra* and + 14% for *Combretum zeyheri* (the only one that increased). Similarly, Mkhize et al. (2018) (South Africa) reported 12 of 14 browse species and 5 of 9 grass species, collected in South Africa, to have greater CT concentrations in the wet season than in the dry season. However, despite this, dietary CT intake rates were significantly greater in the dry than in the wet season, a time when nutrition is likely to be more limiting to ruminant health than parasites.

## Water and heat stress

Water availability is a limiting factor to animal health and productivity, both directly and indirectly through the impacts on the ecosystem. Furthermore, Rockstrom (2000) describes rainfall distribution as a strong driver of livelihood security due to its impact on livestock, crops, and households. The dramatic reduction in rainfall and water availability from the wet to the dry season is therefore a risk factor for livestock systems and the communities they support. For example, Borges (1950) identified water availability to be a major limiting factor to grassland development in Angola. The risk is especially high in the most arid parts of the region such as south-west Botswana and southern Namibia (Peters, 1984). Demand for water is predicted to increase. Masike & Urich (2009) projected that the demand in Botswana for water for livestock may increase by up to 23% due to climate change. Currently, the livestock sector accounts for 75% of agricultural water use in Botswana, which itself accounts for 41% of total water use (Department of Water Affairs, 2015). Notably, the vast majority of water within SoAf agricultural systems is likely to be in the form of green water (Falkenmark & Rockström, 2006; Mafuta, 2018).

Ruminants are far more drought tolerant than most monogastric mammals, due to the rumen acting as a water reservoir. Whilst a > 15% reduction in body mass due to water loss can be fatal for monogastrics, cattle, sheep and goats can easily withstand losses of up to 18% and, in the extreme cases of Boudin goats, up to 40% (Shkolnik et al., 1980), greater even than the 30% loss that camels may tolerate (Schmidt-Nielsen et al., 1956). Ruminants’ ability to replenish their water stocks in a single drinking event is also an important feature of drought tolerance. However, the loss of rumen water to dehydration does have negative impacts on immunity, and fertility, but most importantly for the purpose of this review, on feed intake and digestion. The relationships between water intake, feed intake, and digestion are widely reported and relatively well understood (Chedid et al., 2014; Hadjigeorgiou et al., 2000; Silanikove, 1992). Maloiy et al. (2008) exposed Zebu cattle, Fat-tailed sheep, and Turkana goats to dehydration at 22°C ambient air temperature. Reductions in feed intake were observed for all three species at: −50.0%, −48.0%, and −58.1%, respectively. Reductions were also observed in feed digestion at: −50.5%, −55.8%, and −61.5%, respectively. The effects of heat stress have similar effects to those of dehydration, though the two often come together. Maloiy et al. (2008) (see above) also studied the effects of heat stress and the combined effects of heat stress with dehydration. Under heat-stress conditions (12 h at 22°C then 12h at 40°C) changes in dry matter intake for Zebu cattle, Fat-tailed sheep, and Turkana goats were: −50.0%, + 10.3%, and – 40.5% and changes in digestion were −50.5%, + 3.0%, and −40.5%, respectively. Under conditions of both heat stress and dehydration changes in intake were more extreme at: −63.9%, −59.5%, and −60.5%, respectively, as were changes in digestion which were −69.1%, −64.3%, and −64.4%, respectively. Whilst this is an extreme and experimentally manipulated example, it does highlight the potential impacts of water availability on nutrition. Consequently, the potential of reduced feed intake and utilisation, in response to drought may further exacerbate the impacts of reduced forage nutrition and availability. Whilst droughts are significantly more likely during the dry season, it is a cooler period and thus the risk of heat stress is greatest during the wet season, driven by both high air temperatures and relative humidity.

## Climate change

**Fig. 6:**
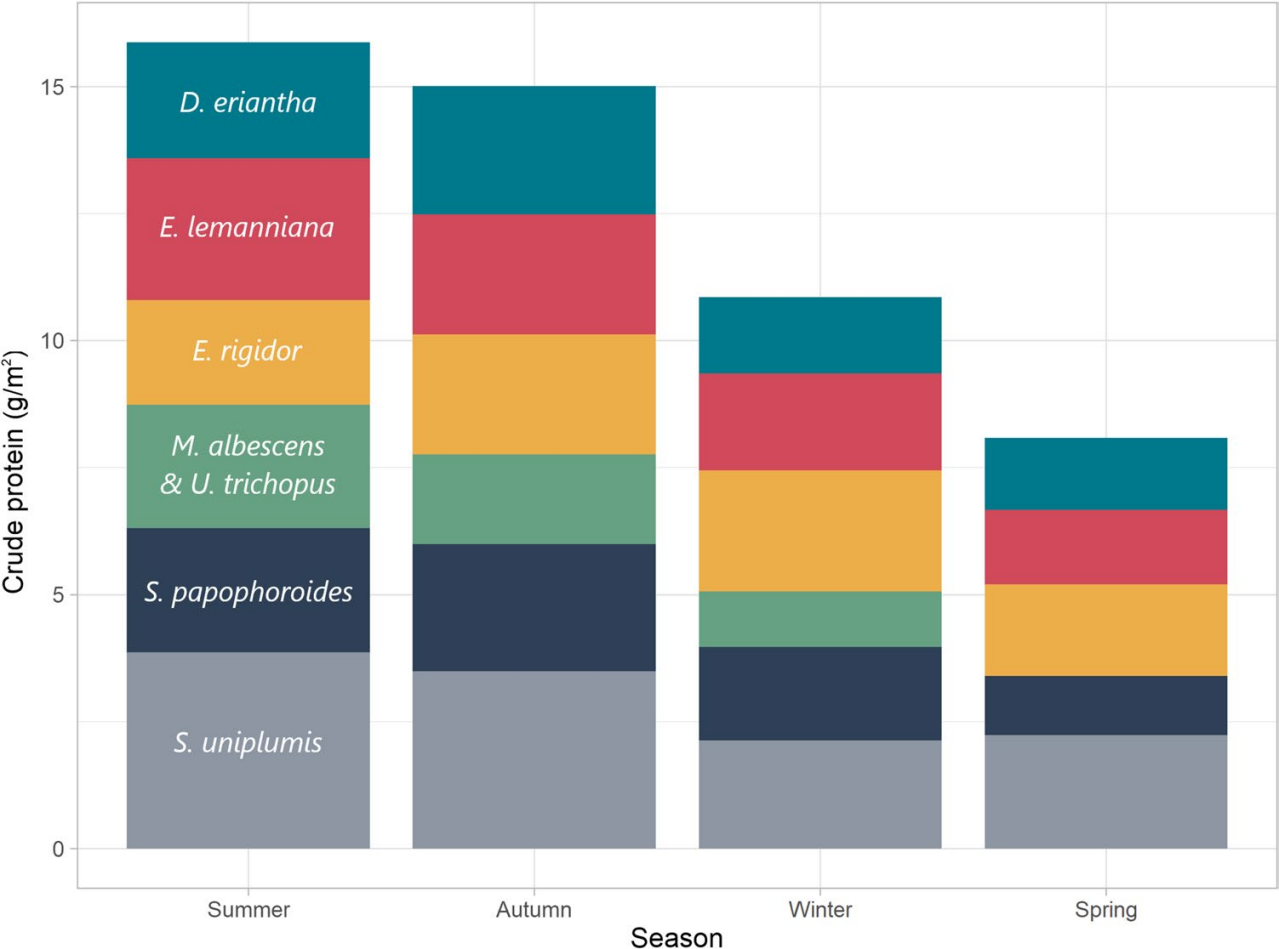
Seasonal changes in crude protein availability across rangeland in Botswana. The Y-axis is crude protein concentration multiplied by biomass (gm2). Megaloprotachne albescens and Urochloa trichopus were combined as per the biomass data in the paper, mean CP concentrations of the two were used. Full species names are Digitaria eriantha, Eragrostis lehmanniana, Eragrostis regidor, Megaloprotachne albescens, Urchloa trichopus, Schmidtia papophoroides, Stipagrostis uniplumis. The wet season occurs in summer. Data taken from Mphinyane et al. (2015)

Food systems across SoAf are threatened by climate change (Masipa, 2017), with the food security of smallholder farmers particularly at risk (Mutengwa et al., 2023). From 1901–1910 the mean annual air temperature across the ten countries was 19.6°C, this rose to 20.3°C in the 1980s, and 20.8°C from 2012–2021 (The World Bank, 2021) (Fig. 6). This trend was particularly pronounced in Lesotho which has seen an increase from 10.6°C (1901–1910) to 12.7°C (2012–2021). Such trends are forecast to continue and SoAf is expected to experience decreasing rainfall, increasing temperatures, and increasingly unpredictable weather (Collins et al., 2013; Davis et al., 2011; Dunning et al., 2018). Lawal et al. (2019) predicted that with 1.5–2.0°C of warming (above pre-industrial levels), ground evaporation will increase over temperate grasslands and there will be increasing incidences of drought. Similarly, Wainwright et al. (2021) forecasts that dry spells in the dry season may be extended by 5–10 days by the end of the century. These factors will lead to a gradual decrease in NDVI as warming continues across Botswana, Mozambique, Namibia, Zambia, and Zimbabwe. However, some parts of South Africa may increase in vegetative productivity. Farmers are aware of these issues and have reported anecdotal evidence of the effects (Hitayezu et al., 2017; Lamega et al., 2021; Makuvaro et al., 2018; Simelton et al., 2013).

The impacts of climate change exacerbate seasonal disparities in forage nutrition, by making either or both seasons less suitable for forage growth and/or by altering the botanical composition of a given system. Currently, higher temperatures and moisture in the wet season facilitate plant growth, but further increases could lessen that effect. For example, as temperatures exceed the mid-high twenties (°C) grass germination rates and seed viability can reduce and this trend is not necessarily linear, with germination rates dropping rapidly as temperatures rise above 30°C (Abdelhak & Mohammed, 2013; Zhang et al., 2012). Whilst the dry season is challenging due to droughts, the lower temperatures may currently be a relief and help to limit evapotranspiration, although this benefit may be lost if temperatures rise, thereby making the season less hospitable. Increased droughts will increase the likelihood of crop failure, reducing the availability of crop residues as supplementary ruminant feeds. Lawal et al. (2019) suggests that changing conditions and events such as wildfires may encourage the expansion of invasive species. *Prosopis juliflora* (which originated in Latin America) has spread across SoAf with negative impacts on ecosystem services and pasture dynamics (Choge et al., 2022). Similarly, invasive grasses have spread within the region (Le Maitre et al., 1996) with largely unknown impacts that might include monopolizing resources (e.g. water and nutrients) from native plants, impacting both biodiversity and available nutrition (LaForgia, 2021).

If forage availability reduces, this may generate competition or conflict. Firstly, competition between species grazing the same land; whilst cattle, goats, and sheep do have dietary niches, there is significant overlap. Secondly, it has been suggested that herder-farmer conflicts may increase due to the pressures of climate change (International Monetary Fund 2022). Elsewhere in Africa, herder-farmer conflicts have been driven by climate-induced perturbations, the absence of protection for grazing lands, and the absence or lack of climate adaptation policies for livestock herders (Tinsley & Gwiriri, 2022). Moreover, the expansion of farming lands into traditional grazing areas, coupled with efforts to sedentarise extensive livestock production systems, further exacerbates the situation. As a consequence, these challenges have led to reduced forage availability and nutritional resources, ultimately intensifying herder-farmer conflicts. Bennett et al. (2007) described the preference of cattle and sheep towards crop residues. If climate change creates a mismatch between forage availability there may be an increased likelihood of free-roaming livestock attempting to consume crops before harvesting, risking the provoking of such conflicts (Cabot, 2017). Should these risks not be recognised and mitigated through effective policies in SoAf, deleterious implications, such as those occurring elsewhere in Africa, may become more prevalent. Planned provision of crop residues in times of nutritional feed gap could conversely serve to increase co-operation and reduce tensions.

## The nutritional feed gap

These combined seasonal effects on nutrition moving from the wet to dry season can be termed a “*Nutritional Feed Gap*” – the rapid and significant reduction in dry matter availability to ruminants, compounded by a similarly significant reduction in forage quality, resulting in an elevated risk to the health and sustainability of ruminant systems. These effects are exacerbated by water availability for animals and will further be exacerbated under the effects of climate change.

Quantifying and understanding the nutritional feed gap crucially relies on data for both forage quantity and quality. However, this is something that was notably lacking in the literature. Research typically investigates one of these two factors independently with very few studies examining the combined impact of both. There is therefore a risk that the literature could underestimate the nutritional feed gap, either previously or in the future. Here, we propose that future research should aim to look beyond nutritional quantity and quality independently but to look at the combined effect. This is particularly pertinent to any form of nutritional budgeting or climate-based forecasting.

**Fig. 7:**
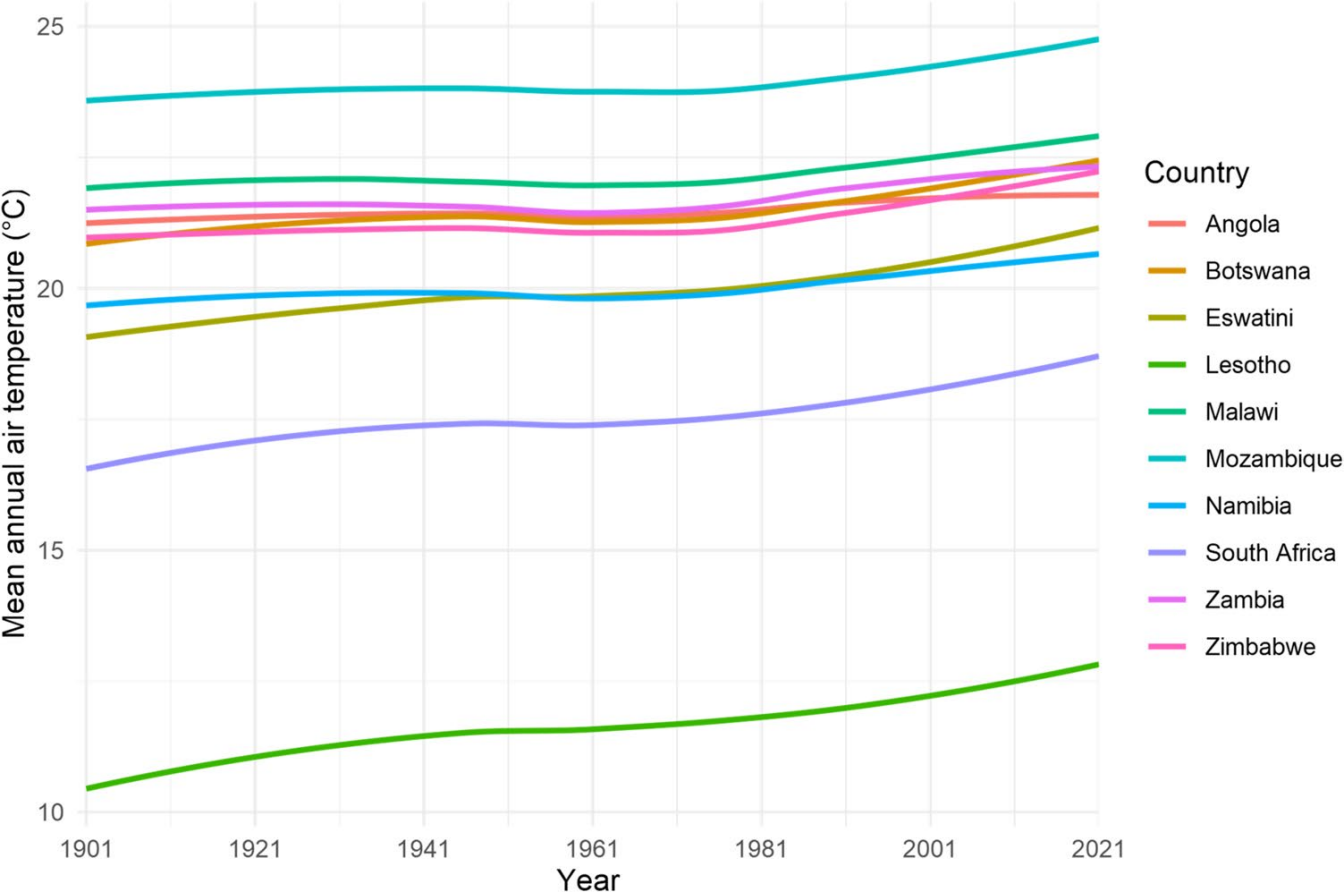
Mean annual temperature of countries within SoAf from 1901 to 2021. Data taken from The World Bank (2021)

One study to combine both was Mphinyane et al. (2015), who reported CP and biomass of grasses in rangeland in Botswana. Although the study presented these variables independently of one another, it provides sufficient data to estimate the combined effect by combining the CP and biomass data from the study. It is thus possible to see the extent of seasonality across seven grass species for the studied rangeland in terms of the total availability of CP per square-metre. Resultant calculations show that during the summer (wet season) CP was 15.9 g/m^2^ which almost halves to 8.1 g/m^2^ in spring (dry season) (Fig. 7). Though a slightly different approach, these results are broadly in line with those by Mataveia (2018) who found wet season CP consumption of goats in Mozambique to be 94.5 g/day during the wet season but only 55.5 g/day in the dry. As is clear by Fig. 7, the impact of reduced crude protein concentration in dry matter is compounded by the reduced availability in overall dry matter.

### Impacts on food security

The significant dietary and financial contribution of ruminants to rural communities means that seasonal risks to ruminant production can also be considered as risks to human food security. Crucially however, the extent of that risk (likelihood and severity) on human food security has not been quantified. Reduced nutritional quantity and quality during the dry season can lead to reduced animal health and performance, which limits the yields and profitability of ruminants. For example, Martins & Peters (1992) reported that lambs born during the dry season had lower birth weights than those born in the wet season and that heavier lambs born in the wet season, struggled to maintain condition through the dry season. Similarly, Nsoso et al. (2003) reported lower body condition scores for Tswana goats in the dry season than in the wet season. Reproductive performance is fundamental to the economic productivity and sustainability of ruminant systems (Abraham et al., 2018) but is significantly impacted by nutrition (Dupont et al., 2014) and thus reproduction is challenged during the dry season. Kraai et al. (2022) reported lower reproductive performance of goats in South Africa in the dry seasons. Similarly, Slayi et al. (2022) reported that goats in their first parity during the dry season were the most at risk of abortions, citing nutrient deficiency as the driving factor. Linking this directly to food security, Airs et al. (2023) reported that higher kid birth likelihoods and a greater number of goats kids were both associated with reduce concern for food security, whilst Kgosikoma et al. (2012) cited poor reproductive performance of cattle, driven by nutritional inadequacy, as a driver of food insecurity in Botswana. Ultimately, smallholders who suffer livestock losses are more likely to be food insecure, whilst those who are able to maintain or improve goat health can see positive impacts on their livelihoods.

It is important to note that the wet season is not without its challenges and numerous studies report high mortality during this period as the warm and moist conditions can be conducive to diseases such as *Haemonchus contortus*, tick-borne pathogens, and foot rot. Ramabu et al. (2024) reported goat and sheep mortality to be greatest in the wet season, citing disease as a key factor, but also inadequate nutrition, possibly driven by conditions in the dry season. Tackling the nutritional feed gap may therefore not only improve performance in the dry season but also support animals to fight the pathogens they may be exposed to in the wet season.

Animal agriculture is just one aspect of human food security and arable agriculture is another significant component. However, seasonal challenges to arable production often coincide with the nutritional feed gap. The ‘lean season’ is a period between harvests where access to arable agricultural products is particularly low, and typically occurs sometime during the dry season. Across sub-Saharan Africa, the lean season has been identified as the time with the lowest household food security, requiring increased needs to buy food, which is at a premium (Becquey et al., 2012; Bonuedi et al., 2022; Sibhatu & Qaim, 2017). This lean season is especially detrimental if it occurs early, as happened in SoAf in 2015/16 due to a drought in the wet season (Rembold et al., 2016). This supports the need for nutritional strategies to improve ruminant performance to help mitigate shocks of the ‘lean season’, especially through the role of ruminants as insurance assets. Whilst this is evidence that the nutritional gap, through its impacts on ruminant production, impacts human food security, detail (especially quantitative) as to the nature and mechanism of that impact is lacking.

## Intervention and mitigation

Addressing the nutritional feed gap is a necessary but complex challenge. Whilst single interventions may have localised or short-term benefits, more comprehensive strategies and investment are needed at local, national, and regional levels to support the long-term food security of farming communities. Commercial systems appear capable of mitigating the impact of the nutritional feed gap and this has been seen somewhat in South Africa. This is usually achieved through any combination of drought management/water conservation, feed supplementation, forage preservation, genetic selection, and veterinary care (Callaghan et al., 2014; Eslamian & Eslamian, 2017; Malau-Aduli et al., 2021). Interventions must also be cognisant of salient ecological dynamics of grazing areas, particularly in dryland ecosystems. Such mitigation is typically reliant on investment, however, this is a limiting factor across small holder systems in SoAf, which are the most food insecure. Nevertheless, opportunities must be taken to identify, research, and fund mitigation strategies to empower rural communities in this endeavour.

### Forage conservation

Forage conservation, such as the creation of silage, hay, or dried pellets could mitigate the nutritional feed gap in the dry season and help farmers cope with unexpected or exceptional events (e.g. droughts). However, forage preservation is challenging for smallholders due to a lack of resources (Balehegn et al., 2022; Chakoma, 2012). Furthermore, spoiled forage is not only an economic loss, but may be a threat to animal health (e.g., mycotoxins in silage) (Ogunade et al., 2018). Balehegn et al. (2022) discussed the challenges of forage conservation in Africa, citing education, resources, land tenure, and market forces as major barriers. Low-cost and low-tech forage preservation methods have been explored (e.g. Manyawu et al., 2016) with some success, but widespread adoption has been poor. Forage conservation does not necessarily require the mass harvesting and storing of forage, but may also include standing forage, which may integrate well with cut-and-carry systems. Apart from silage and supplementary feed from cultivated crop plants (e.g. *Lablab purpureus*), farmers may also be trained to harvest, dry, and store wild nutraceutical plants, some of which are already used in cut and carry systems. Although this may be regionally specific and a laborious solution, it could buffer against nutritional shocks in the dry season. Some harvested species that may be used as fodder for small ruminants include but are not limited to *Acacia* spp (leaves and pods), *Terminalia* spp, *Dichrostachys* spp, *Combretum* spp, *Grewia* spp, *Boscia* spp., and *Colophospermum* spp. (Dambe et al., 2015).

#### Outreach and education

Communication and training must be a high priority if wider uptake of forage conservation is to be achieved. Outreach and extension activities have had mixed success in the region and long-term follow-up is often limited. Whilst researchers typically understand the value of on-farm outreach activities, such as farmer field schools, funding is limited and often short-term, making it difficult to enact sustained change.

Fadeyi et al. (2022) found that formal/school education was positively associated with farmer adoption of new technology/processes. The inclusion of women in both outreach and education is also vitally important, with gender being the second most cited barrier (after finance) to farm innovation. The mainstream inclusion of agricultural science within the school curricula would yield benefits for the next generation of farmers.

The expanse of technology is improving the delivery of information, with Gwiriri et al. (2023) reporting that nearly 90% of goat farmers in Botswana own mobile phones. With veterinary and animal health services often unavailable, unaffordable, or inaccessible, these represent key mechanisms to deliver information and education. Information should include long-term weather forecasts, epidemic warnings, or education in forage nutrition or animal health monitoring techniques. These also provide platforms by which farmers discuss issues, practice, and collaborate. However, ownership and utilisation of a mobile phone may not be sufficient to support improved productivity and instead how a farmer uses their phone is critical (Quandt et al., 2020), suggesting the need for relevant capacity-building interventions to maximise the potential of mobile phone ownership for farmers. Inequities in technology access are present with mobile phone access higher in men and youth (Okano et al., 2022).

#### Land tenure

Food security relies on sustainable and productive agricultural systems and these require investments of time, labour, and resources into the land. However, ruminants typically graze across extensive communal systems and ownership and tenancy of grazing land is uncommon and often on an insecure or short-term basis. For example, in Botswana (1999) the majority of land was considered ‘tribal’ (71%), followed by ‘state’ (25%) compared to little under ‘freehold’ (4%) (Adams et al., 2003). Many smallholders therefore do not have the opportunity to develop grazing systems and thus, the planting of forages and controlled grazing, is uncommon.

In larger and more intensive commercial systems, the planting and conservation of forages are more common due to clear land tenure and simplified decision processes. There are additional and growing pressures on grazing land availability as urban areas expand and as land is turned over for cropping, infrastructure, or other commercial practices. For smallholder farmers, regenerative rangeland management strategies such as rangeland resting, intensive corralling and tightly bunched herding have the potential to increase rangeland biodiversity, grazing capacity and co-existence of cattle and wildlife (Odadi et al., 2011; Young et al., 2018). Active management allows for setting aside of forage resources, for example, to fill expected or unexpected nutritional feed gaps.

#### Market forces

Efficient markets underpin food security by facilitating the transfer of resources, ensuring market access, and providing price stability, which not only helps mitigate short-term issues but also facilitate profitable livestock enterprises. Across SoAf, there is a limited ability to assess forage quality and consequently, the prices for conserved forages do not correlate well with quality (Ayantunde et al., 2021; Jarial et al., 2016). This can inhibit the pursuit of forage conservation as a commercial exercise and notably the improvement of forage quality. From the buyer’s perspective, it also undermines their trust in the product. Therefore, rapid and easily useable predictors of forage quality are needed, such as hand-held near infra-red (NIR) equipment within co-operatives. Linking smallholder farmers to forage seed markets, as well as structured livestock marketing systems might drive investment in interventions that increase productivity. However, price caps that are not determined by market forces may function negatively. In Botswana the price cap for any goat is currently tagged at P1000.00 (⁓US$70.00). It is generally considered ‘unusual’ to sell or buy a goat at a price inconsistent with this. This price is the highest for any small ruminant livestock in the region, and within the country, goats are considered the most expensive livestock. The government uses this price to purchase stock from farmers for youths and other farmers in its various citizen empowerment programs. While farmers selling to the government may profit, this price is arguably too high and unsustainable for the local market relative to beef. Therefore, in times when the government stops buying goats, the demand slumps, resulting in low sales, leading to serious market challenges, and negating the benefits of goat rearing. How this coincides with and compounds/mitigates the nutritional feed gap has not been explored. Unregulated pricing based on market forces may open up local markets, including butcher and supermarket sales channels to promote a more stable and predictable market in the long-term, but may expose consumers and retailers to price and supply shocks.

Finance is the most commonly cited barrier to the adoption of new technologies and processes (Fadeyi et al., 2022). Whilst small-scale forage conservation is possible with little resources (e.g. air drying of hand-harvested forage), producing conserved forages reliably and to scale requires significant capital investment and incurs ongoing costs, which are unfeasible for most smallholders in SoAf. Farmer cooperatives are increasing in popularity and such schemes may bridge this gap.

#### Ecological dynamics

The ecological dynamics of grazing areas must be considered in rangeland management strategies to address nutritional feed gaps, particularly in semi-arid and arid areas. Behnke & Mortimore (2016) discuss how misunderstandings of dryland ecology, driven by the ‘equilibrium ecology’ (EE) assumption (that systems are naturally stable but have been perturbed by human activities such as livestock herding), leads to maladaptive interventions focused on environmental control such as promoting fixed water points or sedentarisation (Behnke & Mortimore, 2016; Scoones, 2018).

Dryland ecosystems are rather governed by non-equilibrium ecology (NEE) dynamics, whereby “ecosystems are often strongly controlled by external forces rather than, or in addition to, internal biotic factors” (Ellis & Swift, 1988). In essence, ‘external forces’ such as anomalously low levels of precipitation often determine degradation in dryland systems, rather than overstocking or overgrazing. Rangeland degradation is unlikely when the coefficient of variation of rainfall is above 33%, as herbivore pressure during droughts is low relative to available levels of biomass (Ellis & Swift, 1988; Illius & O’Connor, 1999). Von Wehrden et al. (2012) employed a statistical literature analysis and found no cases of widespread vegetation change, on spatial scales akin to desertification, in non-equilibrium rangelands. However, below this value, rangeland degradation can occur if animal numbers are high enough which necessitates targeted livestock and rangeland management training. The findings of von Wehrden et al. (2012) were reinvestigated by Engler & von Wehrden (2018) using a geo-referenced database of peer-reviewed studies, finding strong support for the NEE concept for semi-arid and arid rangelands, while notably finding no evidence of systematic differences between commercial and subsistence rangeland farming in terms of biophysical indicators of degradation. The latter point emphasises the need for recognition of NEE dynamics within both commercial and subsistence livestock systems.

Vetter (2005) emphasises the importance of considering both EE and NEE perspectives to produce comprehensive interventions that are more effective and context-specific. For example, drought often causes herbivore numbers to reduce, which recover slowly in natural circumstances. The subsequent reduction of grazing pressure allows for the rangeland’s ecosystem to recover (Ellis & Swift, 1988). However, the introduction of artificial water points and feed supplementation (based on EE understandings of dryland ecosystems) threatens to disrupt this natural NEE cycle (Mortimore & Turner, 2005), risking long-term ecosystem degradation if not combined with rotational management practises that allow for the vegetation to recover (Behnke & Mortimore, 2016). Scoones (2004) emphasises that climate change will shift rangeland ecosystems across Africa to NEE dynamics over increasingly larger areas, and as such it is vitally important that EE-based strategies focused on control, predictability and managerialism are replaced with more people-centred, decentralised approaches that focus upon ecological dynamics, poverty, and livelihoods (Behnke & Mortimore, 2016; Dobie, 2001; Scoones, 2004, 2018).

### Animal selection

Previously, there have been issues with the importation of European genetics, particularly of dairy cattle, into African systems to which they are ill-suited (Eisler et al., 2014). Whilst that has mostly passed, it highlights how appropriate species and breed selection are vital for forage utilisation and hardiness. The popularity of goats in the region, which have a high dietary plasticity and drought tolerance compared to cattle and sheep is fundamental to food security.

The selection of heat and drought-resistant phenotypes is essential to ensure resilience to the increasingly extreme seasonality across SoAf. Whilst indigenous breeds such as Tswana and Boer goats do possess many of those traits (Monau et al., 2018) there has been limited optimisation of those through selection. The picture in cattle is more complex, with *Bos taurus* and *Bos indicus* both present. *Bos indicus* are more tolerant to heat and drought conditions (Beatty et al., 2006) and are a valuable genetic resource both independently or crossed with *Bos taurus*. However, the suitability of cattle in certain locations must be considered under the pressures of climate change, given their relatively low thermal and drought tolerance compared to goat and sheep. Indeed, some have argued that camels may be the most suitable livestock in some scenarios (Faye, 2013, 2014). However, the socio-cultural status of cattle in SoAf, and the rising global demand for beef, may be at odds with this.

### Winter forages

Moyo & Ravhuhali (2023) reported that 94% of surveyed farmers in the East Cape of South Africa found winter forages, primarily oats (*Avena sativa*), beneficial to sheep production, citing improved condition and wool quality. Though, with an average of 17 cattle, 18 goats, and 90 sheep per respondent, results may not represent smallholders or subsistence farmers lacking resources for feed crop cultivation. However, cooperatives can be beneficial in making this more realistic at a community level. The biological feasibility of growing winter forages will vary geographically. Furthermore, in a region with a high incidence of food insecurity, providing human-edible crops to livestock may not always be the most efficient or socially acceptable use of resources. Furthermore, competition for land between arable and livestock farmers has already led to conflict in the region (Darkoh & Mbaiwa, 2009; Mazonde, 1996).

## Knowledge gaps

The primary driver behind ruminant production in SoAf is to deliver nutrition for human consumption, which falls against a backdrop of high levels of food insecurity, malnutrition, and heightened risks of famine in the region. The association of ruminant production with food security is therefore an intrinsic one. Despite this, a key finding of this study is that the impact of the nutritional gap on human food security is poorly understood, perhaps in parts to its indirect nature. There is also a great degree of complexity in that relationship; in addition to animal nutrition and performance factors, climate science, epidemiology, and socio-economics, undoubtedly all play significant roles. There is therefore a need for long-term and multidisciplinary research in this area to better establish the association between the availability of ruminant nutrition and human food security. This must account for a wide variety of scenarios both at the individual level (e.g. financial security) and at regional/international levels (e.g. market dynamics, climate change). As an example, there is a relatively well understood association of financial security with ruminant ownership characteristics (type, number, and use/off-take), but we don’t understand how those two factors influence an individuals´ risk exposure to the nutritional gap, or indeed other external challenges. A better understanding of the complex relationships in these systems will allow for more targeted intervention, more nuanced policy, and better forecasting of the impacts of short and long-term climatic events.

Symptomatic of the limited multi-factorial or multi-disciplinary work in this area, few studies collected data on both forage nutrition and availability, with the majority focussing on the former. Mphinyane et al. (2015) was the only study that truly enabled the characterisation of the nutritional feed gap at a specific site. But even then, the study itself did not quantify the combined effects. This highlights an oversight in the field and limits our ability to fully characterise the nutritional feed gap, let alone the broader implications towards human food security. Where there is sufficient forage availability relative to stocking density, it is the nutritional composition that is of primary concern, however across much of SoAf that is not the case. Furthermore, even where the total abundance of forages is sufficient, a lower density of forage will at least require more energy expenditure by ruminants (and possibly farmers) to meet DMI requirements. One of the simpler and immediate solutions to quantifying forage quantity is the use of open-source remote sensing data (e.g. NDVI) as a proxy.

There was little research available from Angola, Eswatini, and Lesotho. For the latter two, this is likely due to their small size. Information from southern Mozambique and north-eastern South Africa should have reasonable validity to Eswatini. The altitude and weather of Lesotho makes it somewhat of an outlier, despite it being an enclave in South Africa. The lack of literature from Angola was surprising as the country has high human and ruminant populations and a reasonably high GDP for SoAf. The primary language is Portuguese, which may limit research dissemination in an English dominated field, though extensive searches by a Portuguese first-language researcher had limited success and furthermore this was less of an issue for Mozambique. A more likely reason is because Angola spends comparatively less GDP on education (2.0%) and research (0.03%) compared the rest of SoAf (education: mean 5.7%, range 3.3–8.9%; research: mean 0.35%, range 0.05 to 0.62%) (The World Bank, 2018). This highlights the need for increased research activity in Angola and the opportunity to support smallholder communities in the country.

Local and indigenous knowledge, such as ethno-veterinary practices, offer opportunities for valuable insight and collaboration. Bruschi et al. (2017) explored this in south Angola and found extensive knowledge of ethno-veterinary plants used by farmers. Similar findings were reported in the same area by Urso et al, (2016) and in South Africa by Matlebyane et al. (2010). In all of these studies, farmers shared knowledge of the medicinal and productivity benefits of a variety of browse and grass species to improve animal health and performance. A secondary benefit of such approaches may also be improved collaboration between researchers and farming communities.

Inconsistent sampling methods were apparent across studies, especially for browse species. For example, Moleele (1998) examined mixed samples of leaves and twigs, Marius et al. (2021) used just leaves, and Stapelberg et al. (2008) took the last 30 mm of branch tips. Methodological consistency would facilitate cross-comparisons of research and improve the external validity of data.

Limited data on vitamin and amino acid concentrations in forages, possible due to resource constraints, could mask nutrient deficiencies. The prevalence of ‘hidden hunger’ is high in the region and therefore understanding the contribution of ruminants in this regard is of significant interest to public health and food security (Adesogan et al., 2020).

## Conclusion

The distinct seasonality of SoAf creates a ‘nutritional feed gap’ that threatens ruminant production in the region. This feed gap occurs from the wet season to the dry season and is characterised by a significant reduction in quality, quantity, and diversity of forages available for ruminants. This reduces their performance leading to lower yields and lower reproductive success which may then impact the availability of animal products to the owner, community, or broader market. The progression of climate change appears to be exacerbating the issue and is likely to continue for the foreseeable future. Although farmers are familiar with and often reactive to this, they have limited resources to respond and protect themselves from the impacts of exceptional events, which are increasingly frequent. This inevitably poses a risk to household and community food security, however the extent of that impact and risk is not well understood and is a key area for future study. Protecting people, animals, and communities from this requires mitigation through strategies such as forage conservation and animal selection, but ultimately relies on sustainable investment in skills, resources, and infrastructure, as well as on political will power. A step towards leveraging those resources may be to begin quantifying the effect of the nutritional feed gap on human food security.

## Glossary

ADF: acid detergent fibre
ADL: acid detergent lignin
CF: crude fibre
CP: crude protein
CT: crude tannin
DE: digestible energy
DM: dry matter
DMI: dry matter intake
EE: equilibrium ecology
GE: gross energy
GIN: gastrointestinal nematode
ME: metabolisable energy
NEE: non-equilibrium ecology
NDF: neutral detergent fibre
NDVI: Normalised Difference Vegetation Index
OC: organic carbon
SoAf: Southern Africa, defined here as: Angola, Botswana, Eswatini, Lesotho, Namibia, Malawi, Mozambique, South Africa, Zambia, and Zimbabwe
